# Evaluating Rumor Debunking Effectiveness During the COVID-19 Pandemic Crisis: Utilizing User Stance in Comments on Sina Weibo

**DOI:** 10.3389/fpubh.2021.770111

**Published:** 2021-11-30

**Authors:** Xin Wang, Fan Chao, Guang Yu

**Affiliations:** School of Management, Harbin Institute of Technology, Harbin, China

**Keywords:** COVID-19, rumor, false information, stance detection, debunking, effectiveness, social media

## Abstract

**Background:** The spread of rumors related to COVID-19 on social media has posed substantial challenges to public health governance, and thus exposing rumors and curbing their spread quickly and effectively has become an urgent task. This study aimed to assist in formulating effective strategies to debunk rumors and curb their spread on social media.

**Methods:** A total of 2,053 original postings and 100,348 comments that replied to the postings of five false rumors related to COVID-19 (dated from January 20, 2020, to June 28, 2020) belonging to three categories, authoritative, social, and political, on Sina Weibo in China were randomly selected. To study the effectiveness of different debunking methods, a new annotation scheme was proposed that divides debunking methods into six categories: denial, further fact-checking, refutation, person response, organization response, and combination methods. Text classifiers using deep learning methods were built to automatically identify four user stances in comments that replied to debunking postings: supporting, denying, querying, and commenting stances. Then, based on stance responses, a debunking effectiveness index (*DEI*) was developed to measure the effectiveness of different debunking methods.

**Results:** The refutation method with cited evidence has the best debunking effect, whether used alone or in combination with other debunking methods. For the social category of *Car* rumor and political category of *Russia* rumor, using the refutation method alone can achieve the optimal debunking effect. For authoritative rumors, a combination method has the optimal debunking effect, but the most effective combination method requires avoiding the use of a combination of a debunking method where the person or organization defamed by the authoritative rumor responds personally and the refutation method.

**Conclusion:** The findings provide relevant insights into ways to debunk rumors effectively, support crisis management of false information, and take necessary actions in response to rumors amid public health emergencies.

## Introduction

COVID-19 spread globally, severely threatening the lives and health of people, and significantly affecting the economy, education, and daily life in various countries (1–3). The United Nations assessed that the COVID-19 pandemic has wiped out decades of developmental gains (4). One unique aspect of the COVID-19 pandemic has been the over-abundance of information on COVID-19-related topics in media and the internet, where true and false information intertwine; the result has been a disruption of the social order. The World Health Organization (WHO) has flagged this situation as an infodemic (information + epidemic) (5–7), noting that an infodemic is the excessive amount of both accurate and inaccurate health information that can spread misinformation, disinformation, misinformation, and rumors during a health emergency, which can hamper an effective public health response (4–8). The role of social and traditional media (e.g., news, TV programs, newspapers, and other mass media) in spreading disinformation and misinformation in the COVID-19 infodemic has now been recognized in a substantial number of literature reports (9, 10). In addition, online social media platforms can easily “facilitate rapid information sharing and large-scale information cascades,” further exacerbating the issue (11, 12). As Gallotti et al. noted on the verge of the global pandemic emergency, human communication on social media is largely characterized by the production of informational noise and misleading or false information (13).

A paradigm-shifting feature of social media is that any user can produce, access, and disseminate content (14) thereby altering the way people communicate, share, receive, use, and search for both general and health-specific information (15); without any doubt, this has the potential to foster the rapid exchange and diffusion of false information (16). However, studies also demonstrate that social media may be very useful for fighting false information during a public health crisis (15, 17) as these social media platforms can be effective tools for combating and mitigating rumors during public health crises (18). Notably, cognitive psychology research suggests that debunking false information is not an easy task (19). Even clearly corrected false information can continue to influence many users who then intentionally or unintentionally ignore the truth and spread false rumors. Despite considerable efforts to debunk rumors, the desired effect has not been achieved (20). Consequently, debunking false information on social media quickly and effectively and curbing its spread has become an urgent task.

Before commencing this research, brief remarks on the terminology are necessary. Regarding the definition and connotation of fake news, misinformation and disinformation, conspiracy theories, satire, and rumors, scholars have different understandings, which are often used interchangeably in academic research (21, 22). In this study, owing to the fact that it includes within it broader related concepts, we have retained the term “rumor” for our research objectives (23). We usually understand rumors as “unverified and instrumentally relevant information statements in circulation that arise in the context of ambiguity, danger or potential threat, and that function to help people make sense and manage risk” (24). The term is applied to a piece of information whose veracity at the time of dissemination is unclear, and thus it may spread false information in the absence of verifiable information (25, 26). In this study, we examined rumors, as they do not imply a judgment about the sender's intention or the veracity of the presented information (23).

Given the seemingly increasing trend of rumor proliferation, an increasing number of researchers have pursued effective debunking and remedial methods to alleviate the negative influence of rumors (27). A common strategy is to use certain fact-checking services to debunk rumors by providing authoritative statements that discern the truth from falsehood (27, 28). These fact-checking platforms include FactCheck.org (http://fackcheck.org), TruthOrFiction.com (http://truthorfiction.com), and Sina Community Management Centre (https://service.account.weibo.com), etc. These online resources can present the truth to the public and play a key role in distinguishing between true and false information (29). However, most fact-checking processes require considerable human labor and material resources, that is, they are time-consuming and expensive (30, 31). Another common strategy is based on psychological theories to explore the psychological mechanisms of rumor acceptance and how corrections and debunking messages attempt to cope with them. For example, Lewandowsky et al. analyzed the cognitive factors that often render misinformation resistant to correction and recommended ways in which corrections should be designed, structured, and applied to maximize the impact of the corrections and reduce the spread of false information (32). In recent years, researchers have focused more on this topic in the context of digital media (33). For instance, several studies indicated that online rumor spread can be reduced by logic- and humor-based corrections (34), links citing truth-related evidence (35), and corrections from reputable sources such as official government agencies (36) and knowledgeable users (37).

However, compared with unverified reports, debunking messages are not as widely shared and spread by users on social media (23, 38). Therefore, some researchers have begun to study improving the effectiveness of rumor debunking on social media, focusing on studying the effectiveness of debunking methods. Only a few research papers qualitatively studied the effectiveness of rumor debunking methods (39, 40) Some analyzed the effectiveness of different rumor-control strategies or specific rumor-control cases (40); for example, Kimmel et al. investigated the efficacy of rumor-control marketing tactics (41); Paek et al. investigated the most effective rumor response strategies to control food safety risks (42, 43); Yang et al. focused on the effectiveness analysis of mixed rumor-quelling strategies by modeling the rumor-truth competing process (44). Other studies aimed to identify key influencing factors to organize debunking methods; for example, Li et al. explored the relationship between social media rumor refutation effectiveness and its possible affecting factors and provided practical suggestions to help accelerate the rumor refutation process (40).

In addition, some studies have shown that effective management of rumors depends not only on the choice of debunking methods but also on the evaluation and response of people to different ways of debunking (42). Thus, some researchers have also begun to study how people respond after rumors have been debunked on social media (29), such as emotional reactions (45), changes in attitudes, and perceived credibility (27). Decades of well-researched experiments, meta-analyses, questionnaires, and surveys offer guidelines for rumor correction and debunking, for example, Pal et al. used an online survey to reveal that denials could be crafted to effectively debunk rumors by incorporating salient beliefs (46), Walter et al. used a meta-analysis to evaluate the relative impact of social media interventions designed to correct health-related misinformation (47). However, these efforts require control over the manipulation of debunking methods (e.g., randomly assigning people to different methods or controlling for the results of many alternative explanations), resulting in higher human, financial, and time costs for debunking rumors on social media, and thus, do little to improve the real-time effectiveness of rumor debunking. Furthermore, the advent of big data has brought a large, constant, and rapidly growing amount of data generated by numerous sources; however, the large amount of interaction data generated by social media has not been extensively studied (40). To better utilize the rich, objective, user-generated research data on social media platforms, this study considers COVID-19 rumors that are widely spread on Sina Weibo, a popular social media in China, as a research sample. Moreover, this study investigates the classification of debunking methods; and based on stance responses to debunking postings in user comments, this study also establishes a method to measure the effectiveness of different debunking methods. This study seeks to reveal the implementation effects of different debunking methods in order to optimize debunking methods and to provide an objective basis for improving their effectiveness.

Thus, to resolve the objectives, we developed a new annotation scheme that manually categorized six debunking methods (i.e., denial, further fact-checking, refutation, person response, organization response, and combination methods) to classify the debunking methods used for the posts on Sina Weibo. Then, deep learning algorithms were used to build text classification models to automatically detect the stance responses (i.e., supporting, denying, querying, and commenting stances) to debunking postings in user comments. In addition, based on the results of identifying user stances in comments, we developed an index to measure the effectiveness of rumor debunking. Finally, an empirical study was conducted using five rumors related to COVID-19 as case studies to compare the effectiveness of different debunking methods, and countermeasures for effective monitoring and combating rumors in public health crises were proposed based on the results of the study.

## Methods

### Study Context and Data Collection

Sina Weibo (http://www.weibo.com), often referred to as China's Twitter (http://www.twitter.com), is one of the most influential social network platforms in China (48). In contrast to other social networks such as Facebook, Instagram, and WeChat, communication on Sina Weibo is almost entirely public. As a popular social media platform with a large user base in China, everyone on social media can communicate, share, receive, use, and search for both general and health-specific information (15). Sina Weibo has not only become one of the primary platforms for releasing and propagating various false information amid the public health crisis (48), but also is a particularly effective tool for combating rumors (18). Therefore, quantitative research on the role of debunking posts on Sina Weibo is relevant to enhancing social media rumor debunking effectiveness (40).

In this study, collaborating with the Zhiwei Data Sharing Platform (http://university.zhiweidata.com, hereinafter referred to as Zhiwei Data), we collected data on COVID-19 rumors through the Business Application Programming Interface (API) of Sina Weibo, as follows:

**(a)** Collecting original postings (i.e., non-reposted postings, hereinafter referred to as postings) related to COVID-19 rumors. First, we defined keyword combinations related to rumors using multiple logical relationships (e.g., AND and OR). Then we collected rumor data related to the COVID-19 pandemic dating from January 20, 2020, to June 28, 2020 (these rumors had been confirmed as “false” by authoritative statements). Second, to avoid data selection bias, we randomly selected five sensational rumors from three categories: authority, society, and politics. These five rumors are the five fake news stories with the highest number of comments from January 20, 2020, to June 28, 2020, which have the characteristics of the most widespread dissemination and influence on social media platforms in China during the COVID-19 pandemic. The annotation scheme of three categories was developed through an iterative process of rounds of annotation and evaluation with three researchers (two Ph.D. students and one expert from Zhiwei Data who are experienced in rumor research). Similarly, after manual data denoising and cleaning by three annotators, we randomly selected 2,053 postings related to the following five rumors:
  1. Yansong Bai dialogued with Nanshan Zhong on “News 1 + 1” (*News*). On January 26, 2020, it was rumored on the internet that CCTV news channel “News 1 + 1” broadcasted a special program on COVID-19 at 21:30, in which host Yansong Bai (a renowned anchor) invited Nanshan Zhong (an authoritative respiratory pathologist) to introduce the effective treatments to prevent the COVID-19 pandemic. The truth was that January 26, 2020, was a Sunday and there was no news show “News 1 + 1.”
  2. Materials of Jiangsu province aided medical team of Hubei province were detained (*Jiangsu*). On February 9, 2020, some netizens released false rumors that a “medical team from Jiangsu province arrived at Wuhan (the capital of Hubei Province) airport, its supplies were looted, luggage was lost, and local doctors and nurses were transported in trucks,” which sparked a heated debate on social media. On February 11, @Jiangsu Province Internet Reporting Centre's official microblogging website clarified that the news was not true.
  3. A postgraduate from the Wuhan Institute of Virology was the “Patient Zero” (*PatientZero*). On February 15, 2020, a false rumor was circulated on social media: Yanling Huang (a postgraduate) from Wuhan Institute of Virology (an authoritative research institute affiliated with the Chinese Academy of Sciences) was “Patient Zero” (the first one to contract the disease and thus the one who started spreading the virus) of COVID-19. The truth was that Yanling Huang graduated from the institute in 2015 with a master's degree, and had been working and living in other provinces since graduation, and has not returned to Wuhan, has not been infected by the COVID-19, and was in good health.
  4. Eighty Chinese citizens were abused while being quarantined in Russian Federation (*Russia*). On March 1, 2020, the Chinese Embassy in Russia informed that a rumor claimed that Moscow (the capital of the Russian Federation) police violently enforced the law, mistreated isolated personnel, and took away Chinese citizens for no reason. It was later revealed that the news was not true.
  5. A car owner in Hubei province died of COVID-19 (*Car*). On June 22, 2020, a rumor was spread that a car owner, who had volunteered to help people by delivering them vegetables, had died from COVID-19. The truth was that the car owner was still alive, and the rumor monger was detained for three days.
**(b)** Collecting all comments that replied to 2,053 postings. From the postings obtained as mentioned earlier, we collected comment conversations associated with each posting. To collect comments, we scraped the web page of each posting to retrieve the URL. However, if the URL was missing or invalid, we chose MIDs (a unique ID of each posting on Sina Weibo) to retrieve the comments; consequently, we collected 100,348 comments (either direct replies or nested replies to the postings) that replied to the 2,053 postings.

As the focus of the study is on debunking rumors, all the selected rumors were false (hereinafter false rumor referred to as rumor) and debunked while the COVID-19 pandemic was still spread. A detailed description of rumors about COVID-19 is shown in Table 1, including the category of rumors, the coding scheme for each category of rumor, and the number of postings and comments of each rumor. In addition, because we used publicly available data, we only referred to the summarized results and did not derive any sensitive information.

**Table 1:** Detailed description of rumors about COVID-19.

**Category of rumor**	**Coding scheme for category of rumor**	**Postings**	**Comments**
Authority	Those slandered by the rumors are authoritative individuals or organizations in expertise fields related COVID-19, such as Nanshan Zhong (*News* rumor), the Wuhan Institute of Virology (*PatientZero* rumor), respectively.		
*News* rumor		478	11,207
*PatientZero* rumor		676	49,763
Society	The rumors about social events, social problems, and social style involving people's daily life about COVID-19, especially reflecting social morality and ethics.		
*Jiangsu* rumor		114	8,006
*Car* rumor		527	15,848
Politics	The rumors related to COVID-19 about politics, i.e., the activities of classes, parties, social groups, and individuals in domestic and international relations.		
*Russia* rumor		258	15,524

*The data collection of postings and comments ended on September 12, 2020, 23:59:59*.

### Annotation Scheme for Debunking Methods

#### Filtering of Debunking Postings

We used manual annotation to filter out postings that debunked rumors. A three-person expert panel of social media researchers (two Ph.D. students and one expert from Zhiwei Data, who are experienced in research on rumor amid public health crisis) labeled the debunking postings out of the 2,053 postings. Referring to Tian et al. (49), the specific steps are as follows:

First, according to the supporting, denying, querying, or commenting (SDQC) stance (annotation rules, see section Stance Classification in Comments That Replied to Debunking Postings), two annotators with a detailed understanding of rumors independently labeled all 2,053 postings.

Second, to eliminate the differences due to human factors, the two members discussed all the annotation results and re-annotated the postings to reach an agreement on the differences.

Third, the third annotator annotated 2,053 postings to calculate inter-rater reliability. Cohen's kappa (κ) for the annotators was 0.921 (95% confidence interval (CI) [0.856, 0.986], *p* < 0.001), indicating a good agreement among them (50, 51).

Finally, from the 2,053 postings, we obtained 1,721 postings of labeled debunking rumors.

#### Categorization of Debunking Methods

The decision as to what debunking methods should be defined, we mainly considered the psychological mechanisms of rumor acceptance and how corrections attempt to cope with them, such as rumor content is sufficiently important (i.e., personally involving or relevant) to people (52), the need for individuals and organizations to combat rumors, and the effect difference between “refutation” and “denial” in debunking rumors (53). In addition, to categorize the debunking methods as broadly as possible, we also referred to some categorization standards described by previous studies:

**(a)** Methods of providing evidence to debunk rumors (38, 54): first-hand experience, URL providing the evidence, quotation by a person or organization, image attachment, quotation by an unverifiable source, reasoning, and without evidence.
**(b)** Rumor-control strategies: refutation, denial, attacking the attacker (42).
**(c)** Debunking response strategies: tweet deletion, rumor clarification with a new tweet, and neither deletion nor clarification (29).
**(d)** Coding scheme for rumor-related messages: rumor messages, debunking messages, uncertainty about rumors, uncertainty about debunking messages, and others (23).

**Figure 1:**
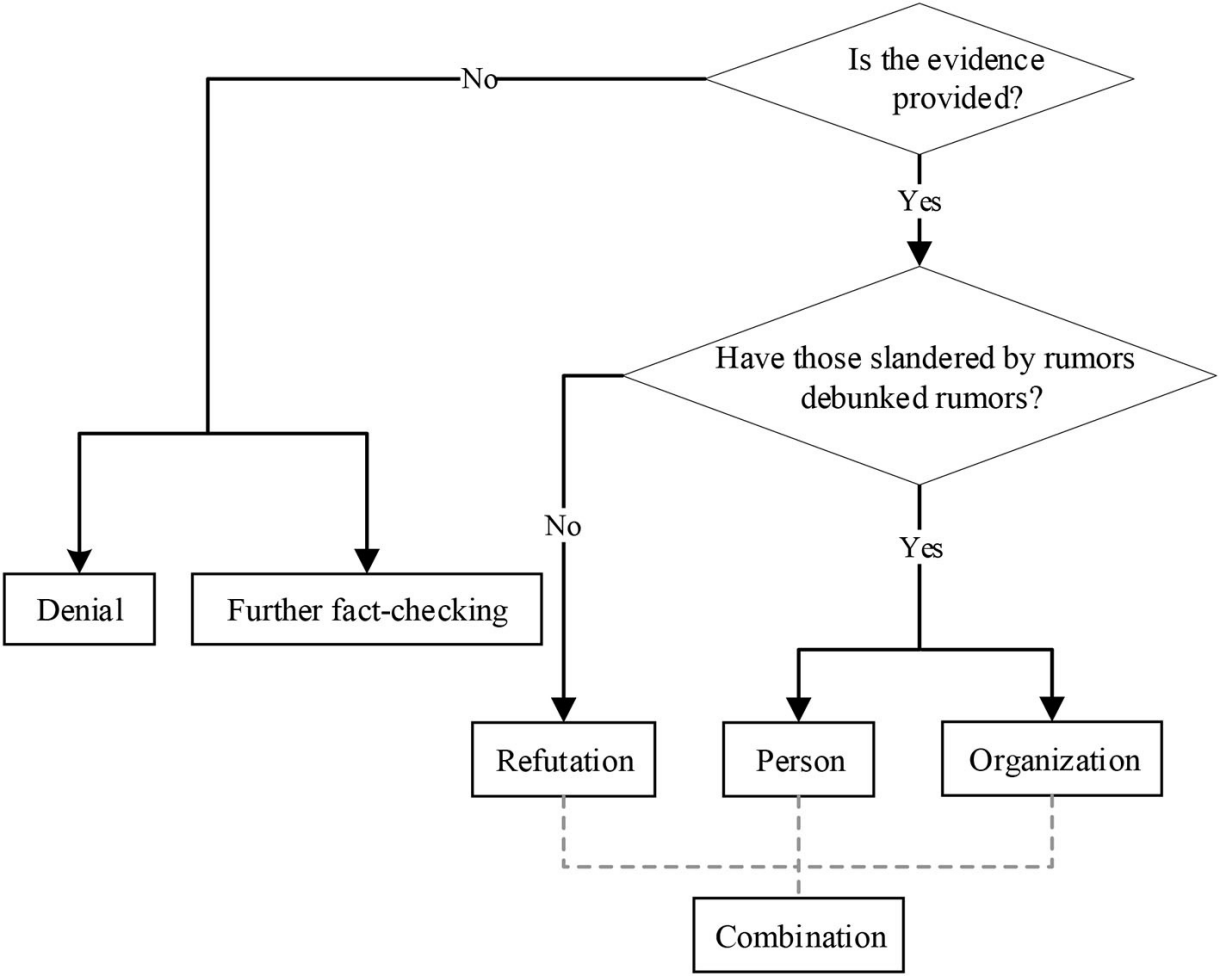
Classification flowchart of debunking methods.

As shown in Figure 1, we designed the classification annotation scheme for the debunking methods as follows.

First, we classified the debunking methods into two main categories: debunking methods with uncited evidence and those with cited evidence. The following are two debunking methods with uncited evidence:

**(a) Denial (Den):** A flat counterstatement of a rumor without providing evidence. E.g., in the *Russia* rumor, “80 Chinese citizens were mistreated in isolation in Russia is ***not true***.”
**(b) Further fact-checking (Fur):** Although people already know the veracity of debunking postings, they still have questions about some details and wish to obtain more relevant information or evidence. For example, in the *News rumor*, “Yansong Bai did not talk with Nanshan Zhong. However, I would want ***the relevant departments do disclose who the rumor monger is*.**”

Second, depending on whether a rumor is debunked by rumors, debunking methods with cited evidence are classified into the following three categories:

**(c) Refutation (Ref):** Those not belonging to those slandered by the rumor provide evidence to debunk rumors. For example, in *the Jiangsu* rumor, the “***Public Security Bureau of Jiangsu Province*** clarified that the news was not true.”

Considering those slandered by the rumor (e.g., persons and organizations slandered by the rumor to debunk rumors personally), another two debunking methods are subcategorized as follows:

**(d) Person response (Per):** Individuals slandered by rumors refute rumors. For example, in the *New* rumor, “Refute the rumor! ***Yansong Bai*** exclusive reply: tonight I did not dialog Nanshan Zhong.”
**(e) Organization response (Org):** Organizations slandered by rumors refute rumors. For example, in *Patient Zero* rumor, “***Wuhan Institute of Virology***: can guarantee that Wuhan Institute of Virology currently has zero infections.”

Third, we defined a combination of debunking methods as follows:

**(f) Combination (Com):** None of the combination methods contain any “debunking methods with uncited evidence” (i.e., Den, and Fur). For example, in the *Car* rumor, “***Hubei Provincial Police*** debunked the rumor; at the same time, ***the car owner's company*** solemnly declared: the car owner is healthy, and the rumors on the online social media are not true.”

Finally, we defined six debunking methods: Den, Fur, Ref, Per, Org, and Com.

Notably, the annotation scheme of these debunking methods was achieved through an iterative annotation process. Similarly, we also asked the three annotators to label the 1,721 debunking postings using the aforementioned annotation scheme. We also assessed the validity of the annotation scheme using Cohen's kappa (κ = 0.747, 95*% CI* [0.676, 0.818], *p* < 0.001), and the results indicated that the annotation process had been validated.

### Stance Classification in Comments That Replied to Debunking Postings

#### Classification Scheme for Comments

With the emergence of rumors on social media, people often express different stances and participate in extensive discussions (29), probably providing more opinions and evidence for rumor detection and debunking. Therefore, mining comments and revealing their SDQC [supporting (S), denying (D), querying (Q), or commenting (C)] stances on a false rumor help determine the veracity of rumors (55, 56), thereby realizing early detection and intervention of rumor spread. SDQC stance classification is an effective approach used by researchers to judge opinions and stances (57). Therefore, utilizing the rich information in comments that replied to the postings on Sina Weibo, we employed SDQC stance classification (38) to classify the stance in comments on a debunking posting into one of the four stances. As shown in Table 2, the comments of each user were categorized using the following four stances (56).

**Table 2:** Coding scheme for four stances (Translated into English from Chinese).

**Stance**	**Description**	**Example**
Supporting (S)	Users who commented to debunking postings believe that a rumor is true, i.e., they think debunking postings is false.	In the *News* rumor, “I'm specifically setting my alarm clock to wait to read about the COVID-19 on “News 1 + 1.” Take precautions, wish for peace, and defeat this epidemic.”
Denying (D)	Users who commented to debunking postings believe that a rumor is false, i.e., they think debunking postings is true.	In the *Russia* rumor, “80 Chinese citizens were mistreated in isolation in Russia is not true.”
Querying (Q)	Users who commented to debunking postings while ask for additional evidence in relation to the veracity of a rumor.	In the *PatientZero* rumor, “I'm a little suspicious, can you continue to investigate? The mentors have come out to clarify, can Huang Yanling come out to speak?”
Commenting (C)	Users who commented to debunking postings not express their clearly stance whether they wanted to assess the veracity of a rumor.	In the *PatientZero* rumor, “What exactly does Patient Zero mean? Patients are counted from “1,” and there is something wrong with counting from 0 itself, right? I thought in the world of mathematics, 0 is not exact.”

#### Stance Classification

To improve the classification efficiency and obtain satisfactory results in the case of massive text data, we designed text classifiers with supervised learning methods to automatically identify and classify the four types of SDQC stances in 100,348 comments.

Similarly, first, we asked two members of our team to label 12,000 comments (randomly selected from 100,348 comments) with the corresponding SDQC stances.

Second, the third member labeled 12,000 comments to validate the inter-annotator agreement, and the annotation process was assessed and validated using Cohen's kappa (κ = 0.876, 95*% CI* [0.856, 0.896], *p* < 0.001).

Third, for the 100,000-level dataset in this research, based on an empirical rule, therefore we had to use a greater percentage of data to develop and test models and used the old way of splitting data to divide the dataset scientifically, that is, the non-redundant 12,000 comments were randomly divided into the independent training set and testing set according to the ratio of 7:3 (58–60). The label distribution is presented in Table 3.

**Table 3:** Label distribution of the training and testing sets.

	**Stance**			
	**Supporting**	**Denying**	**Querying**	**Commenting**
Training set	227	2,305	1,126	4,742
Testing set	98	956	472	2,074

Fourth, we trained and compared the text classifiers using deep learning methods. Because the new language representation model, bidirectional encoder representations from transformers (BERT), developed by Google in 2018, is conceptually simple, empirically powerful, and obtains new state-of-the-art results on 11 natural language processing tasks without substantial task-specific architectural modifications, we chose BERT and its related improved two models to train the classifiers (61). These three models are BERT, RoBERTa-wwm-ext (robust optimized BERT approach-whole-word masking-extended data), and RBT3 (three-layer RoBERTa-wwm-ext) (62–64). The performance of the proposed classifiers was evaluated using the macro-F1 score (used in the multi-class stance classification) (65, 66). Due to the prevalent phenomenon of class imbalance in text classification, the most widely used performance measure for multi-class text classification is the F1 score which is defined as the harmonic mean of precision and recall (67). It is known to be more informative and more useful than classification accuracy etc., especially for multi-class imbalance problems (65, 68). In macro-F1, we used each stance *j* to compute that particular stance's precision *P*_*j*_ as well as recall *R*_*j*_, and finally computed a simple average of the F1 scores over classes (equal weight to each class) to get macro-F1 (65, 68).

During the fine-tuning process, we compared 72 sets of hyperparameters based on the three models to obtain the best-performing stance classification model. Table 4 presents the performances of these classifiers under different combinations of hyperparameters. For brevity, we have only shown the hyperparameter results with the best performance for each model. Detailed results of the stance classification are described in Supplementary Table 1. Finally, we chose the RoBERTa-wwm-ext model with hyperparameters (max_seq_length = 140, train_batch_size = 16, learning_rate = 3e−5, num_train_epochs = 3) to predict the SDQC stance for large comment datasets, as it performed satisfactorily with an accuracy of 80.89% and a macro-F1 of 68.06%.

**Table 4:** Results of stance classification.

**Model**	**Hyperparameters**	**Accuracy**	**Macro-precision**	**Macro-recall**	**Macro-F1**
BERT	(70, 16, 2e−5, 3)	80.33%	67.71%	65.94%	66.51%
RBT3	(140, 32, 5e−5, 3)	80.17%	68.78%	63.08%	64.53%
RoBERTa-wwm-ext	(140, 16, 3e−5, 3)	80.89%	68.76%	67.88%	68.06%

*Hyperparameters (x, y, z, w), where x: max_seq_length (70, 140), y: train_batch_size (16, 32), z: learning_rate (2e−5, 3e−5, 5e−5), and w: num_train_epochs (2, 3). The values in parentheses represent the hyperparameters in the fine-tuning process*.

### Evaluation of the Effectiveness of Debunking Methods

Users' commenting behavior represents their behavioral decisions after they are exposed to postings and process those postings (69). As we used users who commented on debunking postings, we are approximating what the users may have been exposed to regarding debunking postings. Thus, based on stance responses to debunking postings in user comments, this section details the method established to evaluate the effectiveness of different debunking methods.

First, we established a method to evaluate the denial of rumors by users, considering the following two aspects. On the one hand, we considered the stance gap between clear stances in comments, i.e., the stance gap between rumor denial and rumor support. Only a large stance gap between denying stance and supporting stance can effectively show the advantage of denial of rumors by users, i.e., denying stance beats supporting stance, which can exclude the illusion of high denial generated by a high proportion of denying stances and a high proportion of supporting stances as well. On the other hand, we considered clear stances (i.e., supporting and denying stances), which could show the impact of debunking postings, i.e., the validity and persuasiveness of debunking postings in determining truth or falsity, because not all social media users would deny or support rumors even if they had already been debunked by accurate information (29). Thus, referring to the equation by Zubiaga et al. for a simplified analysis of rumor support and denial, they omitted other stances in comments (i.e., querying and commenting stances), which do not contribute to resolving the veracity of a rumor (38). Based on the above analysis, we used the ratio of “the difference between the denying and supporting stances” to “the sum of the two stances, i.e., omitting other stances that do not contribute to resolving the veracity of a rumor from all stances” to calculate the degree of denial [*denial index (DI)*], which could normalize the denying and supporting stances and make *DI* comparable across rumors and events (29, 38). *DI* was calculated as follows:


(1) $$\begin{array}{l} DI_{ij}=\frac{{\#deny}_{ij}-{\#support}_{ij}}{{\#deny}_{ij}+{\#support}_{ij}} \end{array}$$

where *#deny*_*ij*_ and *#support*_*ij*_ denote the number of comments denying and supporting a rumor, respectively, under postings that used debunking method *j* in rumor *i*. In this study, *i* ∈ {1, 2, 3, 4, 5} refers to the *News* rumor, *Jiangsu* rumor, *PatientZero* rumor, *Russia* rumor, and *Car* rumor, respectively, and *j* ∈ {*Den, Fur, Ref, Per, Org, Com*} refers to the debunking method.

Second, to evaluate the effectiveness of different debunking methods accurately, we analyzed the redundancy of users toward different debunking methods; that is, although the users have seen the debunking message, their comments do not contribute toward resolving the veracity of rumors. *The redundancy index (RI)* indicates the extent to which users participate in the process of debunking a rumor. It is defined as “the number of comments with the commenting stance” divided by “the total number of comments.” Accordingly, we have the following:


(2) $$\begin{array}{l} RI_{ij}=\frac{{\#comment}_{ij}}{{\#all\text{\_}stance}_{ij}} \end{array}$$

where *#comment*_*ij*_ and *#all*_*stance*_*ij*_ denote under postings that used the debunking method *j* in rumor *i*, the number of comments in which users did not clearly indicate their stance on the veracity of a rumor and the number of all comments, respectively.

Third, we defined the *debunking effectiveness index (DEI)* to measure the effectiveness of debunking rumors. To comprehensively develop a measurement for the effectiveness of debunking rumors, we considered two aspects. On the one hand, the *DEI* measures the impact of the debunking posting, including two aspects, one aspect is related to the overall number of comments, i.e., examining whether the debunking posting is more influential, such that it receives more comments; Another aspect is related to lower redundancy in comments, such that it receives fewer comments that do not contribute to resolving the veracity of a rumor. Thus, the impact of the debunking posting was measured by *RI*. On the other hand, the *DEI* measures the denial of debunking postings and, similarly, includes two aspects. One measures whether the denying stances get more comments than supporting stances; the other measures for the validity and persuasiveness of debunking postings in determining truth or falsity, i.e., a simplified analysis of rumor support and denial to normalize the denying and supporting stances. Thus, the denial of the debunking posting was measured by *DI*. Generally, in comments in which users respond to a debunking method, if the number of comments with the denying stance is sufficiently higher than that of comments with irrelevant information that does not contribute to the veracity of rumors, then using the debunking method, users can clearly express their denial of fake news without expressing too many redundant or irrelevant comments. Therefore, this way of debunking might be satisfactory in disseminating true information and debunking rumors. Conversely, we considered that *DEI* was directly proportional to *DI* and inversely proportional to *RI*. Given these two values, *DEI* was calculated as follows:


(3) $$\begin{array}{l} DEI_{ij}=\frac{DI_{ij}}{RI_{ij}} \end{array}$$

where *DI*_*ij*_ and *RI*_*ij*_ denote the degree of user denial and the redundancy of comments, respectively, when debunking method *j* is used in rumor *i*.

**Figure 2:**
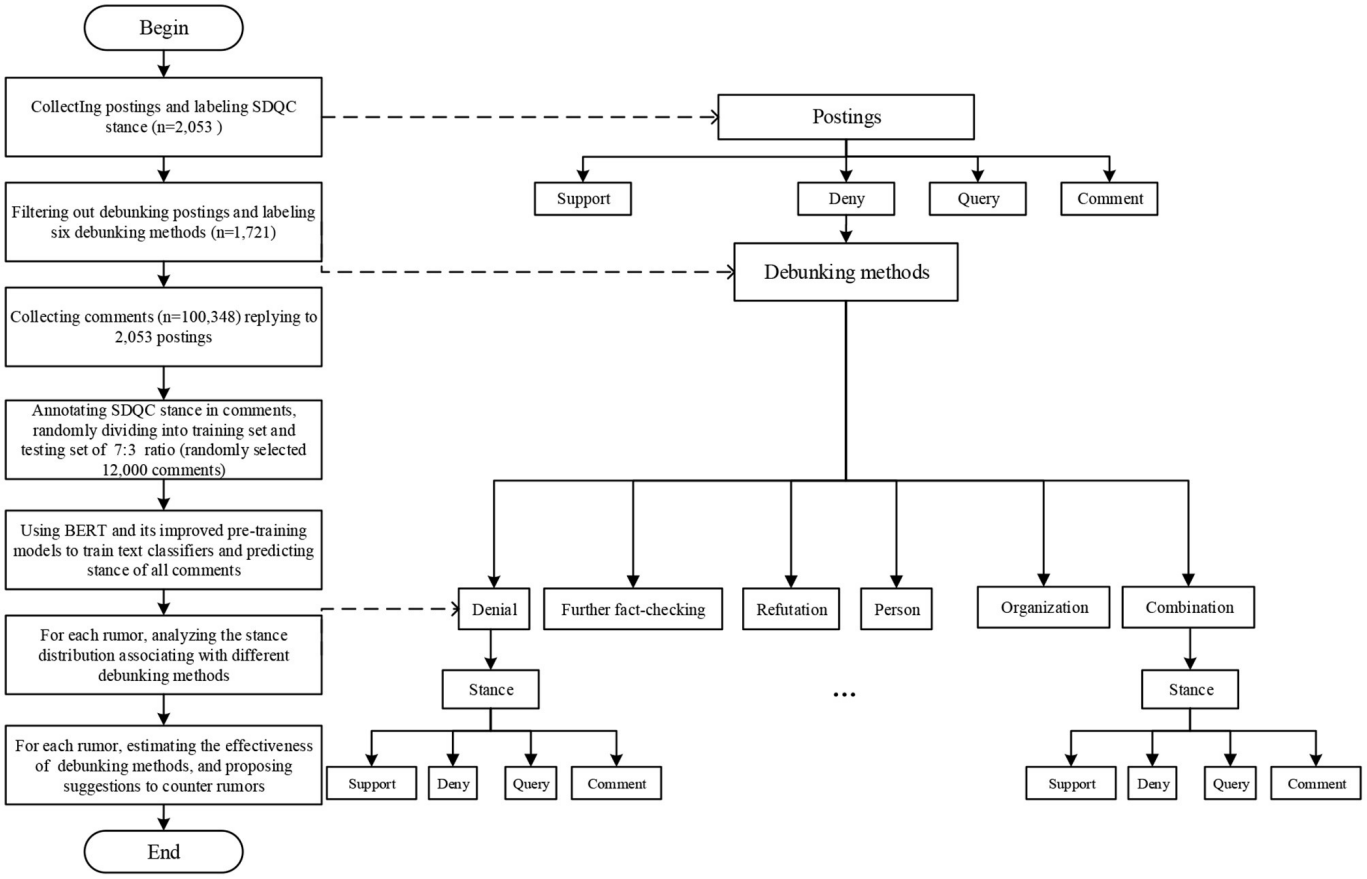
Technology roadmap.

Figure 2 shows the technology roadmap of the overall research process. The flowchart on the left side of the dotted line describes the overall research process; the tree diagram on the right side of the dotted line represents the data annotation related to the research process.

### Statistical Analysis

Descriptive analysis of the basic information of the rumors was conducted using SPSS for Windows, version 25.0.0 (IBM Corporation). The Pearson chi-square (χ^2^) tests were performed to compare the difference in posting distribution of each debunking method across five rumors, as well as the differences in SDQC stance distribution under different debunking methods across five rumors (70). The Kruskal–Wallis tests were applied to evaluate whether there were statistically significant differences in the distribution of *DIs*, *RI*s, and *DEIs* under different debunking methods for each rumor (71). The *post-hoc* test with Bonferroni correction was used for pairwise comparisons when the overall test was statistically significant (72). In addition, we adopted a more general view of the *p*-value as a statistical summary of the compatibility between the observed data and what we would predict or expect to see if we knew the entire statistical model (all the assumptions used to compute the *p*-value) were correct (73, 74). Thus, the *p*-value can be cautiously interpreted as a continuous measure of the compatibility between the data and the entire model used to compute it, ranging from 0 for complete incompatibility to 1 for perfect compatibility, and in this sense may be viewed as measuring the fit of the model to the data (74).

## Results

### Distribution of Debunking Postings

**Figure 3:**
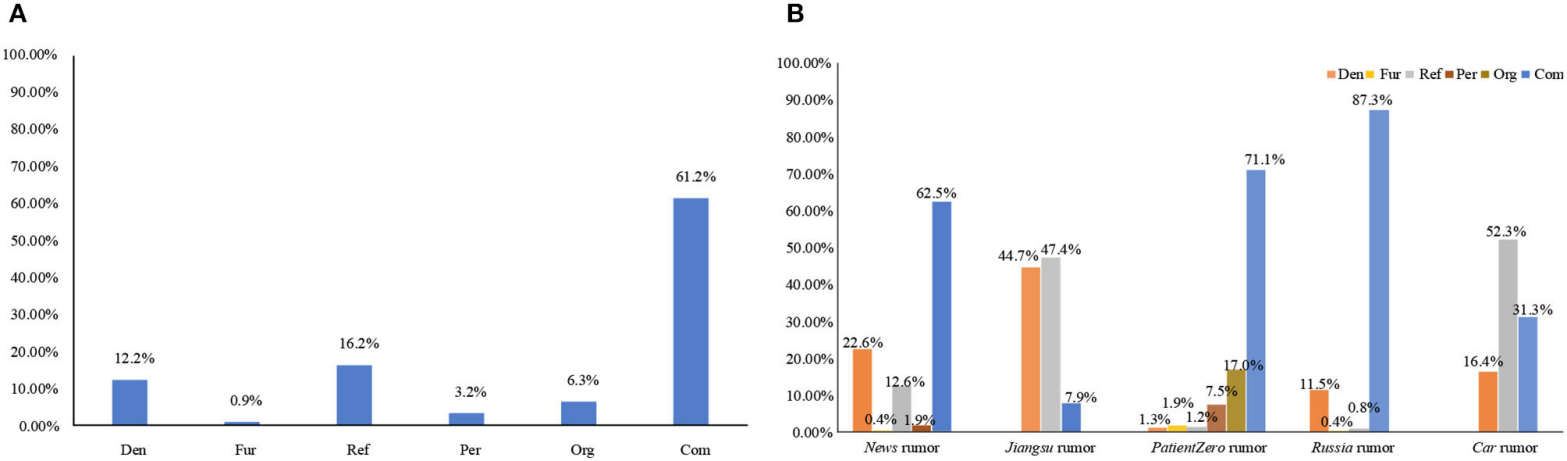
Proportion of postings of the six debunking methods in the **(A)** overall and **(B)** each rumor. Den, Denial; Fur, Further fact-checking; Ref, Refutation; Per, Person response; Org, Organization response; Com, Combination.

Figure 3 shows the proportion of postings for each debunking method, indicating the frequency of use of different debunking methods. The result of the Pearson Chi-square test was χ^2^(25) = 958.273, *p* < 0.001, indicating that the result had a very high degree of statistical significance. Generally, as shown in Figure 3A, more than half of the methods were used to debunk rumors through a combination of methods (Com), with a percentage of 61.2%, considerably exceeding the respective proportion of other debunking methods, followed by the refutation method (Ref, 16.2%) and denial method (Den, 12.2%).

Additionally, for each rumor, we analyzed the proportion distribution of postings that used different debunking methods. In Figure 3B, we observed the following: the combination method was most commonly used in the *Russia* rumor (Com, 87.3%); the refutation method was most commonly used in *Car* rumor (Ref, 52.3%); the denial method was most commonly used in the *Jiangsu* rumor (Den, 44.7%). The debunking methods that employed organizational (Org) and personal (Per) responses were most used in *the PatientZero* rumor, with percentages of 17.0 and 7.5%, respectively. However, the further fact-checking method (Fur) was used less frequently in all rumors, with a maximum of 1.9% in *PatientZero* rumor.

### Stance Classification in Comments That Replied to Debunking Postings

**Figure 4:**
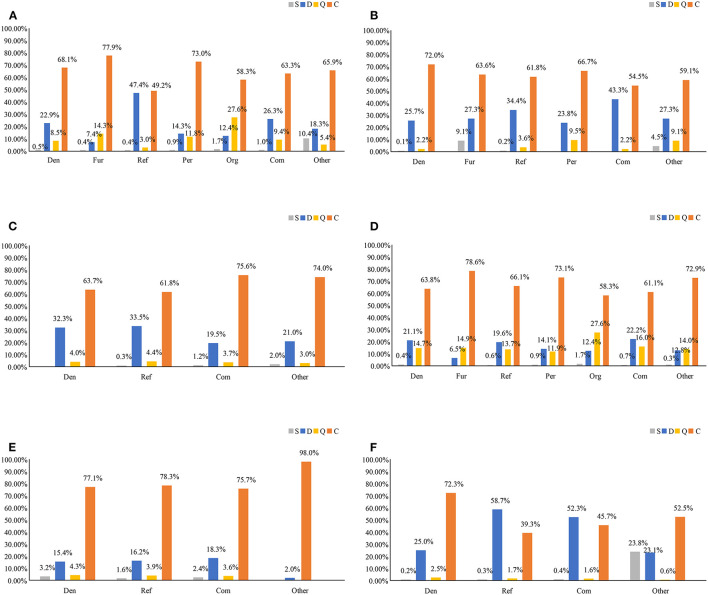
Comparison of the number and proportion of comments for the SDQC stances corresponding to different debunking methods in **(A)** overall, **(B)** *News* rumor, **(C)** *Jiangsu* rumor, **(D)** *PatientZero* rumor, **(E)** *Russia* rumor, and **(F)** *Car* rumor. Other: all non-debunking methods with SDQC stances on rumors.

We also investigated the SDQC stance distribution in the comments of users who participated in different debunking methods. The results of our stance classifier are shown in Figure 4; they include the number and proportion of comments for each SDQC stance under various debunking methods. For five rumors, Pearson Chi-square tests indicated that the results had a very high degree of statistical significance in the SDQC stance distribution under different debunking methods [Figure 4A: χ^2^(30) = 19733.495, *p* < 0.001; Figure 4B: χ^2^(15) = 299.396, *p* < 0.001; Figure 4C: χ^2^(9) = 80.294, *p* < 0.001; Figure 4D: χ^2^(18) = 1649.622, *p* < 0.001; Figure 4E: χ^2^(9) = 32.584, *p* < 0.001; Figure 4F: χ^2^(9) = 3335.866, *p* < 0.001].

From Figure 4A, it is evident that overall, compared with the debunking postings, the supporting (S) stance accounted for a greater proportion of non-debunking methods, with a percentage of 10.4%. This result shows that postings that use different debunking methods can reduce the impact of rumors. However, the denying (D) stance accounted for a higher percentage for the refutation method (47.4%). Additionally, the querying (Q) stance accounted for a higher proportion for the organization debunking method (Org, 27.6%). The commenting (C) stance accounted for the highest proportion for each debunking method, with a maximum for the further fact-checking method (Fur, 77.9%), indicating that the number of comments that did not contribute to the veracity (i.e., true or false) of a rumor was high on Sina Weibo.

Similarly, as shown in Figures 4B–F, we analyzed the stance classification results for each rumor. As shown in Figure 4F, the supporting (S) stance accounted for the highest proportion of non-debunking methods in *Car* rumor (23.8%). Similarly, the denying (D) stance had the highest proportion for the refutation method in *Car* rumor (Ref, 58.7%). Simultaneously, for each rumor, the proportion of the querying (Q) stance was lower, although it accounted for a relatively higher proportion of the debunking method for the organizational response in *the PatientZero* rumor (Org, 27.6%) (see Figure 4D). Finally, the commenting (C) stance had the highest proportion (98.0%) for non-debunking methods in the *Russia* rumor (see Figure 4E).

### Estimation of the Effectiveness of Debunking Methods

#### Measurement of Debunking Effectiveness

Through the aforementioned analysis results, we can see the differences among various debunking methods, both overall and in each rumor. As shown in Table 5, to analyze the performance of social media users without considering the characteristics of rumors, we describe and analyze the overall performance of denial, redundancy, and debunking effectiveness index (*DI*, *RI*, and *DEI*, respectively). For different debunking methods, Kruskal–Wallis tests indicated that the results had a very high degree of statistical significance in the distribution of *DIs*, *RI*s, and *DEIs* (see Table 5). The results of *post-hoc* comparisons *via* Kruskal–Wallis test with Bonferroni correction were also shown in Table 5.

**Table 5:** Comparison of *DEI* under different debunking methods.

**Debunking method^**a**^**	**DI^**b**^**	**RI**	**DEI**	**Pairs (I, J), Adj. Sig.^**c**^^,^^**d**^**
Den	0.959	0.681	1.409	(Ref, Den), *p* < 0.001; (Ref, Fur), *p* < 0.001; (Ref, Per), *p* < 0.001; (Ref, Org), *p* < 0.001; (Com, Den), *p* < 0.001; (Com, Fur), *p* < 0.001; (Com, Ref), *p* < 0.001; (Com, Per), *p* < 0.001; (Com, Org), *p* < 0.001;
Fur	0.905	0.779	1.161	
Ref	0.986	0.492	2.004	
Per	0.881	0.730	1.207	
Org	0.762	0.583	1.307	
Com	0.925	0.633	1.461	
Mean 95% CI	0.901 [0.837, 0.964]	0.654 [0.605, 0.704]	1.430 [1.238, 1.622]	
Median Chi-Square (df) Sig.	0.925 851.081(5) *p* < 0.001	0.633 709.992(5) *p* < 0.001	1.461 782.305(5) *p* < 0.001	
Kruskal-Wallis				
H Sig.	800.471 *p* < 0.001	827.813 *p* < 0.001	748.546 *p* < 0.001	

a *Den-Denial; Fur-Further fact-checking; Ref-Refutation; Per-Person response; Org-Organization response; Com-Combination*.

b *Most of our rumor data were collected retrospectively after the truth was revealed, which represents the eventual general trend in public opinion as truth-driven, hence explaining the calculated high value of DI in our results*.

c *The Adj. Sig. value was the adjusted p-value, which was employed with a Bonferroni-type adjustment of p-value*.

d *For brevity, we only listed the main post hoc testing results*.

For *DEIs*, the *post-hoc* comparisons *via* Kruskal–Wallis test with Bonferroni correction showed that the results had a very high degree of statistical significance between combination method and other five types of debunking methods, and the results had a very high degree of statistical significance between refutation method and other five types of debunking methods (see Table 5). Thus, the refutation method has the best debunking effect (*DEI*_*Ref*_ = 2.004), followed by the combination method (*DEI*_*Com*_ = 1.461). Obviously, the debunking effects of these two methods considerably exceed those of the other debunking methods. Moreover, compared with using the refutation method alone, the combination method did not achieve a higher debunking effect.

#### Comparison of Debunking Effectiveness Among Rumors

Because we considered different types of rumors, the same debunking method may have different effects on each rumor. Thus, we used *DEI* to measure and compare the differences in debunking effects among different types of rumors. For each rumor, we also used the Kruskal-Wallis tests to determine whether there were statistically significant differences between *DEIs* across different debunking methods (see Table 6). The *post-hoc* comparisons *via* Kruskal–Wallis test with Bonferroni correction showed that the results had a very high degree of statistical significance between combination method and other five types of debunking methods for *PatientZero* rumor; for *News* rumor, the results had a very high degree of statistical significance between combination method and other four types of debunking methods (excepting further fact-checking method); for *Russia* rumor, the results had a very high degree of statistical significance between denial method and other two methods (combination and refutation methods); and for *Car* rumor, the results had a very high degree of statistical significance between refutation method and other two types of debunking methods (see Table 6). Thus, in the *post-hoc* testing results with a very high degree of statistical significance in Table 6, for authority rumors, i.e., *News* rumor and *PatientZero* rumor, the combination method was the most effective (e.g., *DEI*_*NewsCom*_ = 1.831 and *DEI*_*PatientZeroCom*_ = 1.535). However, for the social category of *Car* rumor and political category of *Russia* rumor, the refutation method was the most effective (e.g., *DEI*_*CarRef*_ = 2.522 and *DEI*_*RussiaRef*_ = 1.047). Furthermore, for *Jiangsu* rumor in the social category, although the refutation method was the most effective (*DEI*_*JiangsuRef*_ = 1.591), the result had a low degree of statistical significance (*H* = 4.160, *p* = 0.125).

**Table 6:** Comparison of *DEI* among five rumors.

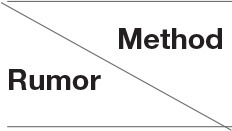	**News**	**Jiangsu**	**PatientZero**	**Russia**	**Car**
Den	1.373	1.571	1.506	0.852	1.355
Fur	0.786	/	1.273	/	/
Ref	1.596	1.591	1.417	1.047	2.522
Per	1.500	/	1.198	/	/
Org	/	/	1.307	/	/
Com	1.831	1.167	1.535	1.019	2.153
Mean 95% CI	1.520 [1.181, 1.860]	1.443 [0.849, 2.037]	1.519 [1.240, 1.798]	0.973 [0.711, 1.235]	1.844 [1.209, 2.479]
Median Chi-Square (df) Sig.	1.831 59.970 (4) *p* < 0.001	1.587 7.500 (2) *p* = 0.024	1.535 25.251 (5) *p* < 0.001	1.019 7.781 (2) *p* = 0.020	2.398 128.749(2) *p* < 0.001
Kruskal-Wallis					
H Sig.	177.256 *p* < 0.001	4.160 *p* = 0.125	228.989 *p* < 0.001	48.002 *p* < 0.001	141.033 *p* < 0.001
Pairs (I, J), Adj. Sig.^a^^,^^b^	(Ref, Den), *p* = 0.033; (Com, Den), *p* < 0.001; (Com, Fur), *p* = 0.159; (Com, Ref), *p* < 0.001; (Com, Org), *p* = 0.037;	/	(Com, Den), *p* = 0.008; (Com, Fur), *p* < 0.001; (Com, Ref), *p* = 0.001; (Com, Per), *p* < 0.001; (Com, Org), *p* < 0.001;	(Com, Den), *p* < 0.001; (Ref, Den), *p* = 0.001; (Ref, Com), *p* = 0.283;	(Den, Com), *p* = 0.001; (Ref, Den), *p* < 0.001; (Ref, Com), *p* < 0.001;

a *The Adj. Sig. value was the adjusted p-value, which was employed with a Bonferroni-type adjustment of p-value*.

b *For brevity, we only listed the main post-hoc testing results*.

#### Analysis of Combination Method

**Figure 5:**
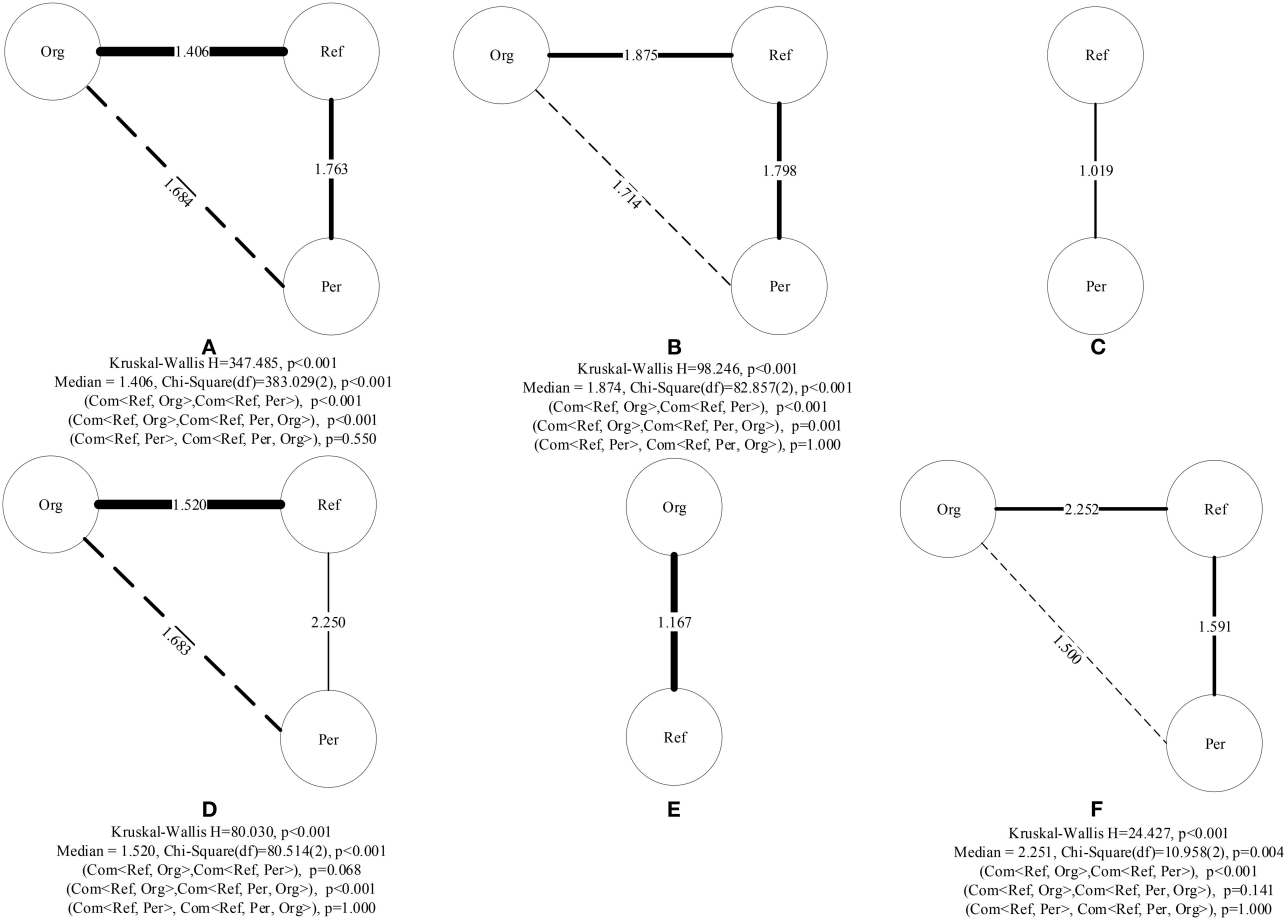
Distribution of debunking methods in a combination method: **(A)** overall, **(B)** *News* rumor, **(C)** *Jiangsu* rumor, **(D)** *PatientZero* rumor, **(E)** *Russia* rumor, and **(F)** *Car* rumor. (a) Although certain combinations of methods are missing in some rumors, our study is based on available data to obtain the best combination of methods to be used for debunking rumors. (b) The *p*-value was the adjusted *p*-value, which was employed with a Bonferroni-type adjustment of *p*-value.

The research results show that the combination method obtains the optimal or suboptimal debunking effect. Therefore, we further analyzed the specific distribution of the combination method. First, we considered decomposing the combination method into five corresponding single debunking methods. In Figure 5, the nodes represent different debunking methods, the edges represent a combination of the respective nodes, and the edge thickness represents the number of comments obtained using this combination method; the thicker the edge, the greater the number of comments obtained in this combination method, whereas the thinner the edge, the smaller the number of comments obtained. In particular, the weight of an edge represents the *DEI* of the combination method. In addition, for different combination methods, Kruskal–Wallis tests indicated that the results had a very high degree of statistical differences in the distribution of *DEIs* (see Figure 5).

For the selected rumors, the combination methods comprise different combinations of three single debunking methods: Ref (a), Per (b), and Org (c). We have used notations such as *Com* < *a, b* > or *Com* < *a, b, c* > to indicate the combination of different debunking methods. Additionally, as shown in Figure 5, the solid-line edge represents the two-tuple *Com* < *a, b* >, whereas the triple-tuple *Com* < *a, b, c* > was formed by the nodes at both ends of the dotted-line edge and their common neighbor node. Meanwhile, the weights represented by each edge are independent, and the weight value on the solid-line edge represents the *DEI* of *Com* < *a, b* >, whereas that on the dotted-line edge represents the *DEI* of *Com* < *a, b, c* >.

First, as shown in Figure 5A, judging from the popularity of comment users, *Com* < *Ref, Org* > as a debunking method received more comments, meaning that using this combination of debunking methods could induce widespread discussion among users. Simultaneously, all the combination methods were combinations of the refutation method and other debunking methods, and *DEIs* of different combinations were relatively different from one another (*H* = 347.485, *p* < 0.001). The *post-hoc* comparisons *via* Kruskal–Wallis test with Bonferroni correction showed that the results had a very high degree of statistical significance between *Com* < *Ref, Org* > and other two types of debunking methods (*Com* < *Ref, Per, Org* > and *Com* < *Ref, Per* >) (see Figure 5). For *Com* < *Ref, Org* > (*DEI*_*Com*<*Ref,Org*>_ = 1.406) and *Com* < *Ref, Per, Org* > (*DEI*_*Com*<*Ref,Per,Org*>_ = 1.684), with the incorporation of one debunking method, *DEI*_*Com*_ increased slightly. However, for *Com* < *Ref, Per* > (*DEI*_*Com*<*Ref,Per*>_ = 1.763) and *Com* < *Ref, Org* > (*DEI*_*Com*<*Ref,Org*>_ = 1.406), the *DEI* of the combination method varies with different combinations of debunking methods. Therefore, the research results show that incorporating another debunking method is not necessarily conducive to improving the debunking effect; however, the scenario needs to be analyzed depending on specific rumors.

Second, for each rumor, the distribution of each combination method is shown in Figures 5B–F. The *post-hoc* comparisons *via* Kruskal–Wallis test with Bonferroni correction showed that the results had a very high degree of statistical significance between *Com* < *Ref, Org* > and other two types of debunking methods (*Com* < *Ref, Per, Org* > and *Com* < *Ref, Per* >) for *News* rumor; for *PatientZero* rumor, the results had a very high degree of statistical significance between *Com* < *Ref, Org* > and *Com* < *Ref, Per, Org* >; and for *Car* rumor, the results had a very high degree of statistical significance between *Com* < *Ref, Org* > and *Com* < *Ref, Per* > (see Figure 5). According to the results in Table 6, for authority rumors (e.g., *News* rumor and *Patient Zero* rumor), the combination method was the most effective in debunking rumors. In particular, as shown in Figure 5B, in the combination method for *News* rumor, *Com* < *Ref, Org* > was the most effective debunking method (*DEI*_*NewsCom*<*Ref,Org*>_ = 1.875), followed by *Com* < *Ref, Per* > (*DEI*_*NewsCom*<*Ref,Per*>_ = 1.798, *H* = 98.246, *p* < 0.001). Additionally, *Com* < *Ref, Per, Org* > had the weakest debunking effect (*DEI*_*NewsCom*<*Ref,Per,Org*>_ = 1.714). However, for *the PatientZero* rumor, as shown in Figure 5D, *Com* < *Ref, Per, Org* > was the most effective for debunking rumors (*DEI*_*PatientZeroCom*<*Ref,Per, Org*>_ = 1.683), followed by *Com* < *Ref, Org* > (*DEI*_*PatientZeroCom*<*Ref, Org*>_ = 1.520, *H* = 80.030, *p* < 0.001). Additionally, in *post-hoc* comparisons, for *Patient Zero* rumor, although *Com* < *Ref, Per* > was the most effective in debunking rumors (*DEI*_*PatientZeroCom*<*Ref, Per*>_ = 2.250), the results had a low degree of statistical significance between Com < Ref, Org> and Com < Ref, Per> (*adjusted p* = 0.068), and between Com < Ref, Per, Org> and Com < Ref, Per> (*adjusted p* = 1.000).

Third, the results in Table 6 show that for the social category of *Car* rumor and political category of *Russia* rumor, although the refutation method was the most effective debunking way, the combination method still achieved a suboptimal debunking effect. Thus, we still considered the decomposition combination method; the distributions of the corresponding combination methods are shown in Figures 5E,F. Interestingly, for the two rumors, the debunking effectiveness of the combination method did not exceed that obtained using the refutation method alone. Our results indicated that using the refutation method alone could achieve satisfactory rumor debunking effects for these two rumors.

Fourth, from Table 6 and Figure 5, it is evident that the same debunking method differs significantly in effectiveness for different rumors. For example, for the *Car* rumor and *Russia* rumor, the debunking effects of the refutation method are also different (for example, *DEI*_*CarRef*_ = 2.522 and *DEI*_*RussiaRef*_ = 1.047). Similarly, the combination method has different debunking effects in *News* rumor (*DEI*_*NewsCom*<*Ref, Org*>_ = 1.875,*DEI*_*NewsCom*<*Ref, Per, Org*>_ = 1.714) and *PatientZero* rumor (*DEI*_*PatientZeroCom*<*Ref,Org*>_ = 1.520, *DEI*_*PatientZeroCom*<*Ref,Per, Org*>_ = 1.683). Generally, in a manner similar to that for the refutation method, the debunking effect for *Car* rumor is better than that for *Russia* rumor. For the same combination method of *Com* < *Ref, Org* > and *Com* < *Ref, Per, Org* >, if an authoritative person is slandered by the rumor, the debunking effect is better than that for the case in which an authoritative organization is slandered by the rumor.

## Discussion

This study offers three key findings, based on the research results. First, our analysis results showed that, among the six commonly used debunking methods, the refutation method with cited evidence is the most effective method for debunking rumors. Both the refutation and combination methods can achieve satisfactory results, and each combination method contains the refutation method. Thus, for each rumor, the refutation method with cited evidence plays a greater role in debunking rumors. This finding is consistent with those in previous research on rumor psychology literature, that is, the refutation method provides evidence that indicates why false information should not be believed (24). A convincing explanation is a must for debunking inaccurate information and preventing further propagation thereof (41). Compared with the debunking method without evidence, there are two main reasons for the better debunking effect of the refutation method with cited evidence: (a) the vividness and persuasiveness of messages (75), and (b) an evidence-providing refutation message may be perceived as more lucid and persuasive than a flat denial (42). For the latter reason, combined with the rumor psychology formula (*Rumor* = *Importance* × *Ambiguity, R* = *I* × *A*) proposed by Allport and Postman in 1947 (76, 77), the more important the rumor information or the more obscure the evidence, the easier it is to spread, meaning that a refutation method with detailed debunking evidence can have a positive effect (78). This shows that when debunking rumors, the government and other authorities must provide persuasive evidence related to the incident to weaken the ambiguity of rumors and help people identify rumors.

Second, there are different best-performing debunking methods for different rumors. For *Car* rumor and *Russia* rumor, using refutation alone can most effectively combat rumors. Compared with *Russia* rumor, we found that the same refutation method has better debunking effects on *Car* rumor. Based on the analysis of the characteristics of the rumor, we believe that the effectiveness of debunking *Russia* rumor is lower because of the following two possible reasons. First, the global adoption of the Internet has accelerated the rapid spread of the political category of *Russia* rumor among countries and regions. Because of misunderstandings in translation and policies, it has become highly difficult to expose rumors. Second, because of factors such as international political relations, geography, and political sensitivity of the event, there are only a few subsequent reports tracking debunking information, thereby increasing the uncertainty and ambiguity around the spread of rumors. This shows that when dealing with the social category of *Car* rumor and political category of *Russia* rumor, it is necessary to provide concise and strong evidence to debunk it while requiring more focus on *Russia* rumor. For the authority category of *News* rumor and *PatientZero* rumor, our results show that the combination method is the most effective for debunking rumors. This may be because of the trust in authoritative persons and organizations, and people may expect more information or evidence from these authoritative sources, no matter these authoritative sources spread true or false news online (79). Especially during the COVID-19 pandemic, the public has expressed a high degree of trust and expectations in authoritative persons and organizations (80), thereby also reflecting the urgent need for information about the latest developments in COVID-19 treatment. Therefore, a combination of multiple debunking methods is more effective in combating such rumors. Additionally, comparing different rumors, our results show that a combination of too many debunking methods may not have a satisfactory effect in deterring the spread of rumors. This may be because a combination of too many debunking methods would interfere with the normal information selection and cognitive analysis performed by people, decreasing the attention and recognition ability of people owing to information overload, thereby increasing the chances of people being misled by rumors (81).

Third, for rumors of the same category, the corresponding debunking strategies must be adopted according to the different characteristics of rumors. In this study, we selected *News* rumor and *PatientZero* rumor, which belong to the same category as authority rumors, and analyzed the combination method that had the optimal debunking effect. First, when using *Com* < *Ref, Org* > and *Com* < *Ref, Per, Org* > to debunk rumors, the debunking effect for the *News* rumor was better than that for the *PatientZero* rumor. This may be because in the *PatientZero* rumor, owing to public dissatisfaction with the delayed treatment of the early COVID-19 epidemic by the Wuhan government and the accumulation of negative emotions, people doubted the abilities of the government or authoritative organizations to deal with the COVID-19 epidemic, thereby weakening the credibility of official institutions. Thus, irrespective of the measures taken, the public will give negative comments, and consequently, the authoritative organizations will fall into the Tacitus trap owing to the loss of credibility (82, 83). Behind the loss of credibility of the government, there is a psychological phenomenon: the public habitually questions the government (84). This kind of habitual questioning is deep thought and is instinctive but persists for a long time; that is, it will continue to exist regardless of the evidence that is later presented. Therefore, to gain the trust of the public and improve their credibility, governments and other authoritative organizations should focus on the construction of public opinion ecology. Second, for the *PatientZero* rumor, using *Com* < *Ref, Per, Org* > to debunk rumors, the debunking effect is better than using *Com* < *Ref, Org* >. This might be because, in the *Patient Zero* rumor, most of the public indicated the tendency of a person (i.e., Yanling Huang) slandered by the rumor to respond, and the combinations using the refutation, organization response, and personal response methods satisfy the public demand, thus achieving a better debunking effect. This suggests that to curb rumors early, governments should focus on the needs of the public, respond on time to their concerns, and provide more support to meet their information needs. Specifically, when an authoritative organization is slandered by a rumor (e.g., the *Patient Zero* rumor), *Com* < *Ref, Per, Org* > is the most effective combination method, and when the authoritative person is slandered by the rumor (e.g., the *News* rumor), *Com* < *Ref, Org* > is the most effective combination method. Therefore, for authoritative rumors, the choice of the most effective debunking method varies depending on those slandered by the rumor (i.e., person or organization) to debunk rumors. In other words, the most effective combination method requires avoiding the use of a combination of a debunking method where the person or organization defamed by the authoritative rumor responds personally and the refutation method.

### Strengths and Limitations

This study is significant on two fronts, both in theory and practice. On the theoretical front, first, this study proposed a new annotation scheme for debunking methods, aiming to propose corresponding debunking response strategies for different rumors, which enriches research related to debunking methods for social media. To the best of our knowledge, this is the first study to establish a comprehensive coding scheme that can be used to categorize debunking methods in debunking postings, particularly on Sina Weibo. Second, this study proposed a novel method to measure the effectiveness of different debunking methods based on stance responses to debunking postings in user comments. To the best of our knowledge, this study is one of the first to develop a measurement for the effectiveness of debunking rumors. Compared with the decades of well-researched experimental, meta-analysis, questionnaire, and survey work done on false rumors and their correction (46, 47), this study uses deep learning methods to develop optimal classifiers to detect user stance in comments and focuses on developing an index to measure the debunking effectiveness based on stance responses to debunking postings in user comments on social media, which is more objective and scientific. On the one hand, the biggest validity of this assessment method is that it can provide relevant organizations with an effective way to utilize the massive amount of objective data from social media to detect users' stances toward rumors or debunking messages in real-time and achieve timely and effective debunking of rumors. On the other hand, this assessment method does not need to control the manipulation of debunking methods in many traditional works (e.g., randomly assigning people to different methods or controlling for many alternative explanations for findings), which makes debunking rumors on social media more cost-effective in terms of labor, money, and time, and increases the effectiveness of debunking. Our findings have enriched the literature on the mechanism of online effective rumor debunking management and intervention from a text mining perspective during the COVID-19 pandemic, which has rarely been researched in the field of information systems and public health.

This study provides three practical implications for news outlet professionals, social platform managers, and Chinese government regulators regarding the use of rebuttals in combating online rumors during the COVID-19 pandemic. First, we would expect media practitioners and news organizations to comply with professional values while debunking rumors, such as publishing well-founded news after fact-checking, quoting trustworthy sources, and providing appropriately detailed evidence. In particular, practitioners should do a detailed verification of the many aspects of information released by authoritative sources. One of the main reasons is when such fake news stories are conveyed by authoritative figures, as in the case of the lab incident in Wuhan while testing an HIV vaccine (79). When this scenario finds the support of authoritative persons or organizations, it becomes popular and quasi-real, even without being supported by data or evidence, feeding off the idea that there is some sort of plot to silence people that are perceived as menacing or challenging the status quo in the scientific community (79). Therefore, regardless of who publishes debunking information, practitioners should do a good job of fact-checking, they can't let their guard down just because it's the word of an authoritative person or organization. Second, social media managers, in the case of information overload, should consciously verify and strictly control information sources and their published information, take up the vital responsibility of providing users with real and high-quality information, and induce a positive public opinion environment. Third, the Chinese government staff must improve the cognitive ability of its citizens to identify rumors and provide support for the early detection and interruption of rumor spreading, such as improving their digital literacy and health literacy (i.e., the ability to obtain, read, understand, and use healthcare information to make appropriate health decisions and follow instructions for treatment) (85). Simultaneously, to enhance the public's “critical thinking ability” and “trust in science,” different debunking methods should be adopted for different rumors, especially for political rumors and rumors slandering authoritative persons or organizations. Additionally, the government should enhance the monitoring of the debunking effectiveness of official communication channels on social networking sites, such as the Government Information page on Sina Weibo, consider the ecological construction of public opinion, and improve its credibility to win the approval of public opinion. Government-affiliated accounts should leverage social platforms to combat online rumors, attend to the needs and intentions of the public, pay attention to public feedback, and formulate effective debunking strategies to mitigate the existence of rumors at the earliest. Finally, the government and authoritative organizations should improve their credibility by appropriately encouraging commentary-based interactions of the public on social media platforms and enhancing public trust and intimacy with the government.

However, this study has some limitations. First, the sample size and the type of social media platform surveyed were limited owing to the threat of the rapidly spreading COVID-19 pandemic. This paper focuses on the study of five rumors related to COVID-19 in the Chinese social media environment, the generalization of the results to other countries and cultures is constrained. Thus, in future relevant studies, better results might be achieved using adequate sample size and multiple platforms and making comparisons between platforms, such as Sina Weibo and Twitter. Second, although we attempted to use the optimal classifier for text classification in this study, some errors cannot be avoided in the text analysis process. On the one hand, future research could improve the accuracy of the classifier by improving the algorithm to classify users. On the other hand, in this study, the performance of classifiers was evaluated using the macro-F1 score, which was more intuitive but gave equal weight to precision and recall and was ineffective regarding true negatives. Thus, future research can use other high-quality evaluation methods at the same time. For example, for an unbalanced classification problem, future work will consider the use of Receiver Operating Characteristic (ROC) and Area Under Curve (AUC) to measure the performance of classification models, a weighted arithmetic mean with weight *p* given to recall and weight (1−*p*) given to precision to express F-measure (67), and measurement methods in the general multiclass case considering connections between the concepts of informedness, markedness, correlation, and significance as well as their intuitive relationships with recall and precision (86). Third, although we considered two aspects of the effectiveness of debunking, we only used one measure to evaluate the effectiveness of debunking and focused on the post-debunking situation. Thus, on the one hand, future work will consider developing multiple measures that can summarize all aspects of effectiveness, for example, adding an aspect that examines the impact of postings from the perspective of social network structure, such as the depth, breadth, and structural virality of debunking postings, aspects of the characteristics and influence of refuters, and the corresponding aspects of rumor denial and information redundancy. On the other hand, we will consider a better measurement of debunking effectiveness from the perspective of causal effects, such as tracking rumors in real-time, and consider the change in veracity status (such as unverified, true, false) at different points of the life cycle of a rumor, to construct a better measurement of debunking effectiveness which depends on both situations before and after debunking. Fourth, rumors appeal to people because they seem to be able to express or gratify their emotional needs, especially the catharsis of negative emotions related to anger, frustration, hatred, and/or anxiety. Therefore, future research should consider combining the user stance expression, subdividing the problems exposed to negative emotions, and investigating its related influencing factors. Finally, as rumors are often short-lived, there is a temporal dimension that seems to tackle further consideration. This study only portrayed and described the overall phenomenon of rumor debunking and did not consider temporality. Thus, future research will consider analyzing the state changes of various stances that evolve at different periods of the life cycle of a rumor, such as when comments are made or how long rumors or debunking messages persist or remain persuasive, and reveal the underlying reasons behind such changes.

## Conclusions

This study proposed a new annotation scheme to categorize the debunking methods of postings on Sina Weibo. We built text classification models to automatically detect stance responses to debunking postings in user comments. Based on the results of identifying user stance in comments, we proposed a new method to measure the effectiveness of different debunking methods. In addition, we used five rumors related to COVID-19 as cases and compared the effectiveness of different debunking methods. Our main findings are as follows: First, the refutation method is the primary choice among debunking methods and has the best debunking effect, whether it is used alone or in combination with other debunking methods. Second, for the social category of *Car* rumor and political category of *Russia* rumor, using the refutation method alone can achieve the optimal debunking effect. Third, for authoritative rumors, a combination method has the optimal debunking effect, but the most effective combination method requires avoiding using a combination of a debunking method where the person or organization defamed by the authoritative rumor responds personally and the refutation method. Furthermore, we believe this study is significant on two fronts. First, we proposed a new scheme for the classification of debunking methods and formulated a novel method to measure the effectiveness of debunking methods by analyzing the stances of users' comments toward different debunking postings. Second, for each rumor, we revealed debunking strategies that could effectively prevent people from spreading rumors. Our research findings provide relevant insights into ways to effectively debunk rumors amid public health emergencies on social media, aiming to support the crisis management of rumors, with a view to taking necessary actions in response to COVID-19 rumor outbreaks.

## Data Availability Statement

The original contributions presented in the study are included in the article/Supplementary Material, further inquiries can be directed to the corresponding author.

## Author Contributions

XW: conceptualization, methodology, software, visualization, formal analysis, data curation, and writing. FC: data curation, methodology, and formal analysis. GY: conceptualization, writing, and funding acquisition.

## Funding

This research was funded by the National Natural Science Foundation of China (Grant No. 72074060).

## Conflict of Interest

The authors declare that the research was conducted in the absence of any commercial or financial relationships that could be construed as a potential conflict of interest.

## Publisher's Note

All claims expressed in this article are solely those of the authors and do not necessarily represent those of their affiliated organizations, or those of the publisher, the editors and the reviewers. Any product that may be evaluated in this article, or claim that may be made by its manufacturer, is not guaranteed or endorsed by the publisher.

## References

[1] World Health Organization. Coronavirus Disease 2019 (COVID-19) Situation Report-51. Geneva: World Health Organization (2020).

[2] Hui HW Zhou CC Lu X Li JR. Spread mechanism and control strategy of social network rumors under the influence of COVID-19. Nonlinear Dyn. (2020) 101:1933–49. 10.1007/s11071-020-05842-w 32836821 PMC7416597

[3] Romer D Jamieson KH. Conspiracy theories as barriers to controlling the spread of COVID-19 in the US. Soc Sci Med. (2020) 263:8. 10.1016/j.socscimed.2020.113356 32967786 PMC7502362

[4] World Health Organization. Coronavirus Disease 2019 (COVID-19) Situation Report- 45. Geneva: World Health Organization (2020).

[5] World Health Organization. Coronavirus Disease 2019 (COVID-19) Situation Report-13. Geneva: World Health Organization (2020).

[6] Nazar S Pieters T. Plandemic revisited: a product of planned disinformation amplifying the COVID-19 “infodemic.” Front Public Health. (2021) 9:649930. 10.3389/fpubh.2021.649930 34336759 PMC8318131

[7] Banerjee D Meena K. COVID-19 as an “Infodemic” in public health: critical role of the social media. Front Public Health. (2021) 9:231. 10.3389/fpubh.2021.610623 33816415 PMC8012664

[8] Gradoń KT Hołyst JA Moy WR Sienkiewicz J Suchecki K. Countering misinformation: a multidisciplinary approach. Big Data Society. (2021) 8:20539517211013848. 10.1177/20539517211013848

[9] Rovetta A Castaldo L. Influence of mass media on Italian web users during the COVID-19 pandemic: infodemiological analysis. JMIRx Med. (2021) 2:e32233. 10.2196/32233 34842858 PMC8601032

[10] Anwar A Malik M Raees V Anwar A. Role of mass media and public health communications in the COVID-19 pandemic. Cureus. (2020) 12:e10453. 10.7759/cureus.10453 33072461 PMC7557800

[11] Ali K Li C Zaffar MA. Fake news on Facebook: examining the impact of heuristic cues on perceived credibility and sharing intention. Internet Res. (2021). 10.1108/INTR-10-2019-044. [Epub ahead of print].

[12] Vosoughi S Roy D Aral S. The spread of true and false news online. Science. (2018) 359:1146–51. 10.1126/science.aap9559 29590045

[13] Gallotti R Valle F Castaldo N Sacco P De Domenico M. Assessing the risks of ‘infodemics' in response to COVID-19 epidemics. Nature Human Behav. (2020) 4:1285–93. 10.1038/s41562-020-00994-6 33122812

[14] Bessi A Coletto M Davidescu GA Scala A Caldarelli G Quattrociocchi W. Science vs conspiracy: collective narratives in the age of misinformation. PLoS ONE. (2015) 10:e0118093. 10.1371/journal.pone.0118093 25706981 PMC4338055

[15] Alvarez-Galvez J Suarez-Lledo V Rojas-Garcia A. Determinants of infodemics during disease outbreaks: a systematic review. Front Public Health. (2021) 9:236. 10.3389/fpubh.2021.603603 33855006 PMC8039137

[16] Wang YX McKee M Torbica A Stuckler D. Systematic literature review on the spread of health-related misinformation on social media. Soc Sci Med. (2019) 240:12. 10.1016/j.socscimed.2019.112552 31561111 PMC7117034

[17] Vos SC Buckner MM. Social media messages in an emerging health crisis: tweeting bird flu. J Health Commun. (2016) 21:301–8. 10.1080/10810730.2015.1064495 26192209

[18] Househ M. Communicating Ebola through social media and electronic news media outlets: a cross-sectional study. Health Informatics J. (2016) 22:470–8. 10.1177/1460458214568037 25656678

[19] MacFarlane D Tay LQ Hurlstone MJ Ecker UK. Refuting spurious COVID-19 treatment claims reduces demand and misinformation sharing. J Appl Res Mem Cogn. (2021) 10:248–58. 10.1016/j.jarmac.2020.12.005 33391983 PMC7771267

[20] Moravec PL Minas RK Dennis AR. Fake news on social media: people believe what they want to believe when it makes no sense at all. Mis Q. (2019) 43:18–87. 10.25300/misq/2019/15505

[21] Wang Q Yang X Xi W. Effects of group arguments on rumor belief and transmission in online communities: an information cascade and group polarization perspective. Inf Manag. (2018) 55:441–9. 10.1016/j.im.2017.10.004

[22] Wang R He Y Xu J Zhang H. Fake news or bad news? Toward an emotion-driven cognitive dissonance model of misinformation diffusion. Asian J Commun. (2020) 30:317–42. 10.1080/01292986.2020.1811737

[23] Jung A-K Ross B Stieglitz S. Caution: rumors ahead—a case study on the debunking of false information on Twitter. Big Data Society. (2020) 7:2053951720980127. 10.1177/2053951720980127

[24] DiFonzo N Bordia P. Rumor Psychology: Social and Organizational Approaches. Washington, DC: American Psychological Association (2007). 10.1037/11503-000

[25] Dunn HB Allen CA (editors). Rumors, urban legends and internet hoaxes. In: Proceedings of the Annual Meeting of the Association of Collegiate Marketing Educators. Dallas, TX (2005).

[26] Spiro ES Fitzhugh S Sutton J Pierski N Greczek M Butts CT (editors). Rumoring during extreme events: a case study of Deepwater Horizon 2010. In: Proceedings of the 4th Annual ACM Web Science Conference. (2012). p. 275–83. 10.1145/2380718.2380754

[27] Jang JW Lee EJ Shin SY. What debunking of misinformation does and doesn't. Cyberpsychol Behav Soc Netw. (2019) 22:423–7. 10.1089/cyber.2018.0608 31135182

[28] Garrett RK. Troubling consequences of online political rumoring. Hum Commun Res. (2011) 37:255–74. 10.1111/j.1468-2958.2010.01401.x

[29] Wang BR Zhuang J. Rumor response, debunking response, and decision makings of misinformed Twitter users during disasters. Natural Hazards. (2018) 93:1145–62. 10.1007/s11069-018-3344-6

[30] Zhang XC Ghorbani AA. An overview of online fake news: Characterization, detection, and discussion. Inf Process Manag. (2020) 57:26. 10.1016/j.ipm.2019.03.004

[31] Shu K Sliva A Wang S Tang J Liu H. Fake news detection on social media: a data mining perspective. Acm Sigkdd Explorations Newsletter. (2017) 19:22–36. 10.1145/3137597.3137600

[32] Lewandowsky S Ecker UKH Seifert CM Schwarz N Cook J. Misinformation and its correction: continued influence and successful debiasing. Psychol Sci Public Interest. (2012) 13:106–31. 10.1177/1529100612451018 26173286

[33] Malhotra P. A relationship-centered and culturally informed approach to studying misinformation on COVID-19. Soc Med Soc. (2020) 6:4. 10.1177/2056305120948224 34192033 PMC7417961

[34] Vraga EK Kim SC Cook J. Testing logic-based and humor-based corrections for science, health, and political misinformation on social media. J Broadcast Electron Media. (2019) 63:393–414. 10.1080/08838151.2019.1653102

[35] Vraga EK Bode L. I do not believe you: how providing a source corrects health misperceptions across social media platforms. Info Commun Soc. (2018) 21:1337–53. 10.1080/1369118x.2017.131388319088921

[36] Vraga EK Bode L. Using expert sources to correct health misinformation in social media. Sci Commun. (2017) 39:621–45. 10.1177/107554701773177634007225

[37] Alexander DE. Social media in disaster risk reduction and crisis management. Sci Eng Ethics. (2014) 20:717–33. 10.1007/s11948-013-9502-z 24306994

[38] Zubiaga A Liakata M Procter R Hoi GWS Tolmie P. Analysing how people orient to and spread rumours in social media by looking at conversational threads. PLoS ONE. (2016) 11:29. 10.1371/journal.pone.0150989 26943909 PMC4778911

[39] Ozturk P Li H Sakamoto Y (editors). Combating rumor spread on social media: the effectiveness of refutation and warning. In: 2015 48th Hawaii International Conference on System Sciences. Kauai (2015). p. 2406–14. 10.1109/HICSS.2015.288

[40] Li Z Zhang Q Du X Ma Y Wang S. Social media rumor refutation effectiveness: evaluation, modelling and enhancement. Inf Process Manag. (2021) 58:102420. 10.1016/j.ipm.2020.102420

[41] Kimmel AJ Audrain-Pontevia A-F. Analysis of commercial rumors from the perspective of marketing managers: rumor prevalence, effects, and control tactics. J Market Commun. (2010) 16:239–53. 10.1080/13527260902884433

[42] Paek HJ Hove T. Mediating and moderating roles of trust in government in effective risk rumor management: a test case of radiation-contaminated seafood in South Korea. Risk Anal. (2019) 39:2653–67. 10.1111/risa.13377 31294485

[43] Paek H-J Hove T. Effective strategies for responding to rumors about risks: the case of radiation-contaminated food in South Korea. Public Relat Rev. (2019) 45:101762. 10.1016/j.pubrev.2019.02.006 32288053 PMC7126673

[44] Yang L-X Zhang T Yang X Wu Y Tang YY. Effectiveness analysis of a mixed rumor-quelling strategy. J Franklin Inst. (2018) 355:8079–105. 10.1016/j.jfranklin.2018.07.040

[45] Lee J Kanthawala S Britt BC Deavours DF Ott-Fulmore T. Prevalence of anger, engaged in sadness: engagement in misinformation, correction, and emotional tweets during mass shootings. Online Inf Rev. (2021). 10.1108/OIR-03-2021-0121. [Epub ahead of print].

[46] Pal A Chua AY Goh DH-L. Debunking rumors on social media: the use of denials. Comput Human Behav. (2019) 96:110–22. 10.1016/j.chb.2019.02.022

[47] Walter N Brooks JJ Saucier CJ Suresh S. Evaluating the impact of attempts to correct health misinformation on social media: a meta-analysis. Health Commun. (2020) 1–9. 10.1080/10410236.2020.1794553 32762260

[48] Rodríguez CP Carballido BV Redondo-Sama G Guo M Ramis M Flecha R. False news around COVID-19 circulated less on Sina Weibo than on Twitter. How to overcome false information? Int Multidisc J Social Sci. (2020) 9:107–28. 10.17583/rimcis.2020.5386

[49] Tian X Yu G He F. An analysis of sleep complaints on Sina Weibo. Comput Human Behav. (2016) 62:230–5. 10.1016/j.chb.2016.04.014

[50] Cohen J. A coefficient of agreement for nominal scales. Educ Psychol Meas. (1960) 20:37–46. 10.1177/001316446002000104

[51] Reichenheim ME. Confidence intervals for the kappa statistic. Stata J. (2004) 4:421–8. 10.1177/1536867X0400400404

[52] Rosnow RL. Inside rumor: a personal journey. Am Psychol. (1991) 46:484. 10.1037/0003-066X.46.5.484

[53] Bordia P DiFonzo N Haines R Chaseling E. Rumors denials as persuasive messages: Effects of personal relevance, source, and message characteristics 1. J Appl Soc Psychol. (2005) 35:1301–31. 10.1111/j.1559-1816.2005.tb02172.x

[54] Zubiaga A Liakata M Procter R Bontcheva K Tolmie PAcm. Crowdsourcing the annotation of rumourous conversations in social media. In: Proceedings of 2015 International Conference on World Wide Web. (2015). p. 347–53. 10.1145/2740908.2743052

[55] Dungs S Aker A Fuhr N Bontcheva K editors. Can rumour stance alone predict veracity. In: International Conference on Computational Linguistics. (2018). p. 3360–70.

[56] Derczynski L Bontcheva K Liakata M Procter R Hoi GWS Zubiaga A (editors). SemEval-2017 task 8: rumoureval: determining rumour veracity and support for rumours. Meeting of the association for computational linguistics. arXiv [Preprint] arXiv:1704.05972. (2017)

[57] Zubiaga A Kochkina E Liakata M Procter R Lukasik M Bontcheva K. Discourse-aware rumour stance classification in social media using sequential classifiers. Inf Process Manag. (2018) 54:273–90. 10.1016/j.ipm.2017.11.009

[58] Zhou S Ng ST Lee SH Xu FJ Yang YA. domain knowledge incorporated text mining approach for capturing user needs on BIM applications. Eng Constr Architect Manag. (2019) 27:458–82. 10.1108/ECAM-02-2019-0097

[59] Wang P Pei X Yin X-P Ren J-L Wang Y Ma L-Y. Radiomics models based on enhanced computed tomography to distinguish clear cell from non-clear cell renal cell carcinomas. Sci Rep. (2021) 11:1–8. 10.1038/s41598-021-93069-z 34215760 PMC8253856

[60] Lv H Dao F-Y Guan Z-X Yang H Li Y-W Lin H. Deep-Kcr: accurate detection of lysine crotonylation sites using deep learning method. Brief Bioinf. (2021) 22:bbaa255. 10.1093/bib/bbaa255 33099604

[61] Devlin J Chang M-W Lee K Toutanova K. Bert: pre-training of deep bidirectional transformers for language understanding. arXiv [Preprint]. (2018) arXiv:181004805.

[62] Liu Y Ott M Goyal N Du J Joshi M Chen D. Roberta: a robustly optimized bert pretraining approach. arXiv [Preprint]. (2019) arXiv:190711692.

[63] Cui Y Che W Liu T Qin B Wang S Hu G. Revisiting pre-trained models for chinese natural language processing. arXiv [Preprint]. (2020) arXiv:200413922.

[64] Cui Y Che W Liu T Qin B Yang Z Wang S. Pre-training with whole word masking for chinese bert. arXiv [Preprint]. (2019) arXiv:190608101.

[65] Zhang D Wang J Zhao X editors. Estimating the uncertainty of average F1 scores. In: Proceedings of the 2015 International Conference on The Theory of Information Retrieval. New York, NY (2015). 10.1145/2808194.2809488

[66] Asch VV. Macro-and Micro-Averaged Evaluation Measures. Antwerp: Univercity of Antwerp. (2013).

[67] Hand D Christen P. A note on using the F-measure for evaluating record linkage algorithms. StCom. (2018) 28:539–47. 10.1007/s11222-017-9746-6

[68] Yang Y Liu X editors. A re-examination of text categorization methods. In: Proceedings of the 22nd Annual International ACM SIGIR Conference on Research and Development in Information Retrieval. Berkeley, CA (1999). p. 42–9.

[69] Sharpe D. Chi-square test is statistically significant: Now what? Pract Assess Res Eval. (2015) 20:8. 10.7275/tbfa-x148

[70] Kruskal WH Wallis WA. Use of ranks in one-criterion variance analysis. J Am Stat Assoc. (1952) 47:583–621. 10.1080/01621459.1952.10483441

[71] Katz BM McSweeney M. A multivariate Kruskal-Wallis test with *post hoc* procedures. Multivariate Behav Res. (1980) 15:281–97. 10.1207/s15327906mbr1503_4 26794183

[72] Amrhein V Korner-Nievergelt F Roth T. The earth is flat (p> 005): significance thresholds and the crisis of unreplicable research. PeerJ. (2017) 5:e3544. 10.7717/peerj.3544 28698825 PMC5502092

[73] Greenland S Senn SJ Rothman KJ Carlin JB Poole C Goodman SN. Statistical tests, P values, confidence intervals, and power: a guide to misinterpretations. Eur J Epidemiol. (2016) 31:337–50. 10.1007/s10654-016-0149-3 27209009 PMC4877414

[74] Iyer ES Debevec K. Origin of rumor and tone of message in rumor quelling strategies. Psychol Market. (1991) 8:161–75.

[75] Allport GW Postman L. An analysis of rumor. Public Opin Q. (1946) 10:501–17. 10.1093/poq/10.4.501

[76] Allport GW Postman L. The Psychology of Rumor. Washington, DC: Henry Holt (1947).

[77] Chan MPS Jones CR Jamieson KH Albarracin D. Debunking: a meta-analysis of the psychological efficacy of messages countering misinformation. Psychol Sci. (2017) 28:1531–46. 10.1177/0956797617714579 28895452 PMC5673564

[78] Moscadelli A Albora G Biamonte MA Giorgetti D Innocenzio M Paoli S. Fake news and covid-19 in Italy: results of a quantitative observational study. Int J Environ Res Public Health. (2020) 17:5850. 10.3390/ijerph17165850 32806772 PMC7459609

[79] Falcone R Sapienza A. How COVID-19 changed the information needs of Italian citizens. Int J Environ Res Public Health. (2020) 17:19. 10.3390/ijerph17196988 32987914 PMC7579097

[80] Rubin VL. Disinformation and misinformation triangle A conceptual model for “fake news” epidemic, causal factors and interventions. J Doc. (2019) 75:1013–34. 10.1108/jd-12-2018-0209

[81] Giddens A. Risk and responsibility. Mod L Rev. (1999) 62:1. 10.1111/1468-2230.00188

[82] Horn E. Logics of political secrecy. Theory Cult Soc. (2011) 28:103–22. 10.1177/026327641142458317536154

[83] Chen S Yang J. Cultivation of government's credibility in internet age and analyzation of tacitus effect. In: Proceedings of 2014 International Conference on Public Administration. Vol. 10. (2014). p. 347–54.

[84] Sørensen K Pelikan JM Röthlin F Ganahl K Slonska Z Doyle G. Health literacy in Europe: comparative results of the European health literacy survey (HLS-EU). Eur J Public Health. (2015) 25:1053–8. 10.1093/eurpub/ckv043 25843827 PMC4668324

[85] Powers DM. Evaluation: from precision, recall and F-measure to ROC, informedness, markedness and correlation. arXiv [Preprint]. (2020) arXiv:201016061.

