# Supplementary material for: Evaluating Rumor Debunking Effectiveness During the COVID-19 Pandemic Crisis: Utilizing User Stance in Comments on Sina Weibo
Source: Front Public Health. 2021 Nov 30;9:770111. doi: 10.3389/fpubh.2021.770111 (PMC8678741; doi:10.3389/fpubh.2021.770111)
Supplement: Supplementary file 1 [file Table_1.docx]

Supplementary Material

**Supplementary Table 1.** The Performance Evaluation Results.

| **Model** | **Hyperparameters** | **Accuracy** | **Macro-Precision** | **Macro-Recall** | **Macro-F1** |
| --- | --- | --- | --- | --- | --- |
| BERT | (70,16,2e-5,2) | 79.97% | 65.05% | 60.69% | 61.31% |
|  | (70,16,2e-5,3) | 80.33% | 67.71% | 65.94% | 66.51% |
|  | (70,16,3e-5,2) | 80.97% | 72.21% | 62.33% | 64.12% |
|  | (70,16,3e-5,3) | 79.89% | 64.98% | 62.28% | 63.06% |
|  | (70,16,5e-5,2) | 77.67% | 80.53% | 53.87% | 54.84% |
|  | (70,16,5e-5,3) | 79.75% | 66.06% | 62.91% | 63.78% |
|  | (70,32,2e-5,2) | 80.58% | 71.52% | 62.51% | 64.14% |
|  | (70,32,2e-5,3) | 80.94% | 69.75% | 63.75% | 65.25% |
|  | (70,32,3e-5,2) | 79.69% | 68.84% | 60.96% | 62.22% |
|  | (70,32,3e-5,3) | 79.89% | 65.13% | 63.23% | 63.91% |
|  | (70,32,5e-5,2) | 79.29% | 73.23% | 59.76% | 60.16% |
|  | (70,32,5e-5,3) | 79.81% | 64.51% | 62.35% | 63.12% |
|  | (140,16,2e-5,2) | 80.75% | 67.38% | 62.32% | 62.97% |
|  | (140,16,2e-5,3) | 80.61% | 67.24% | 65.70% | 66.09% |
|  | (140,16,3e-5,2) | 79.94% | 73.38% | 61.19% | 62.47% |
|  | (140,16,3e-5,3) | 79.89% | 67.26% | 63.22% | 64.02% |
|  | (140,16,5e-5,2) | 80.33% | 57.25% | 57.97% | 57.57% |
|  | (140,16,5e-5,3) | 80.28% | 67.37% | 66.19% | 66.46% |
|  | (140,32,2e-5,2) | 80.78% | 67.61% | 61.73% | 62.65% |
|  | (140,32,2e-5,3) | 80.39% | 67.41% | 64.47% | 65.07% |
|  | (140,32,3e-5,2) | 80.53% | 73.20% | 61.02% | 62.33% |
|  | (140,32,3e-5,3) | 81.33% | 70.69% | 62.05% | 63.25% |
|  | (140,32,5e-5,2) | 79.03% | 55.97% | 56.03% | 55.85% |
|  | (140,32,5e-5,3) | 80.25% | 73.58% | 60.39% | 61.18% |
| RBT3 | (70,16,2e-5,2) | 79.50% | 81.14% | 57.65% | 57.91% |
|  | (70,16,2e-5,3) | 79.53% | 69.41% | 59.13% | 59.78% |
|  | (70,16,3e-5,2) | 79.47% | 70.38% | 58.87% | 60.01% |
|  | (70,16,3e-5,3) | 78.94% | 64.82% | 62.01% | 62.83% |
|  | (70,16,5e-5,2) | 79.44% | 67.85% | 59.83% | 60.77% |
|  | (70,16,5e-5,3) | 78.83% | 64.30% | 62.25% | 62.98% |
|  | (70,32,2e-5,2) | 78.58% | 80.34% | 57.28% | 57.32% |
|  | (70,32,2e-5,3) | 78.44% | 61.83% | 58.18% | 58.29% |
|  | (70,32,3e-5,2) | 80.19% | 70.74% | 58.50% | 59.32% |
|  | (70,32,3e-5,3) | 79.11% | 63.82% | 59.58% | 60.22% |
|  | (70,32,5e-5,2) | 79.25% | 63.77% | 59.10% | 59.88% |
|  | (70,32,5e-5,3) | 79.31% | 66.41% | 62.43% | 63.64% |
|  | (140,16,2e-5,2) | 79.92% | 76.29% | 58.96% | 59.02% |
|  | (140,16,2e-5,3) | 79.22% | 68.03% | 59.65% | 59.76% |
|  | (140,16,3e-5,2) | 79.56% | 68.57% | 58.34% | 58.83% |
|  | (140,16,3e-5,3) | 79.72% | 65.50% | 61.78% | 62.44% |
|  | (140,16,5e-5,2) | 79.72% | 74.29% | 60.20% | 60.75% |
|  | (140,16,5e-5,3) | 80.19% | 67.17% | 62.64% | 63.70% |
|  | (140,32,2e-5,2) | 79.64% | 68.98% | 58.27% | 58.65% |
|  | (140,32,2e-5,3) | 80.11% | 67.08% | 60.08% | 60.70% |
|  | (140,32,3e-5,2) | 79.64% | 68.70% | 59.63% | 60.81% |
|  | (140,32,3e-5,3) | 79.83% | 65.85% | 63.24% | 64.18% |
|  | (140,32,5e-5,2) | 80.36% | 66.84% | 60.08% | 61.08% |
|  | (140,32,5e-5,3) | 80.17% | 68.78% | 63.08% | 64.53% |
| RoBERTa-wwm-ext | (70,16,2e-5,2) | 80.86% | 72.02% | 62.79% | 64.03% |
|  | (70,16,2e-5,3) | 81.00% | 70.01% | 66.44% | 67.57% |
|  | (70,16,3e-5,2) | 81.03% | 70.35% | 62.78% | 64.24% |
|  | (70,16,3e-5,3) | 80.61% | 67.48% | 64.77% | 65.51% |
|  | (70,16,5e-5,2) | 80.39% | 66.87% | 63.53% | 64.38% |
|  | (70,16,5e-5,3) | 79.64% | 66.84% | 62.84% | 64.10% |
|  | (70,32,2e-5,2) | 81.11% | 72.89% | 64.99% | 66.85% |
|  | (70,32,2e-5,3) | 80.50% | 68.02% | 65.16% | 66.01% |
|  | (70,32,3e-5,2) | 81.11% | 72.78% | 64.72% | 66.20% |
|  | (70,32,3e-5,3) | 81.14% | 69.29% | 65.91% | 66.98% |
|  | (70,32,5e-5,2) | 80.28% | 68.95% | 61.94% | 62.90% |
|  | (70,32,5e-5,3) | 80.61% | 68.45% | 64.79% | 65.80% |
|  | (140,16,2e-5,2) | 81.25% | 71.11% | 63.76% | 64.70% |
|  | (140,16,2e-5,3) | 81.19% | 69.37% | 67.26% | 68.00% |
|  | (140,16,3e-5,2) | 80.75% | 69.86% | 63.08% | 63.94% |
|  | (140,16,3e-5,3) | 80.89% | 68.76% | 67.88% | 68.06% |
|  | (140,16,5e-5,2) | 80.81% | 66.27% | 61.20% | 61.85% |
|  | (140,16,5e-5,3) | 80.58% | 68.36% | 66.66% | 67.17% |
|  | (140,32,2e-5,2) | 81.31% | 71.60% | 63.90% | 65.06% |
|  | (140,32,2e-5,3) | 81.42% | 70.75% | 65.09% | 66.20% |
|  | (140,32,3e-5,2) | 81.25% | 71.92% | 64.14% | 65.58% |
|  | (140,32,3e-5,3) | 81.06% | 68.50% | 65.38% | 66.20% |
|  | (140,32,5e-5,2) | 81.36% | 68.40% | 62.67% | 63.24% |
|  | (140,32,5e-5,3) | 80.33% | 66.70% | 65.14% | 65.67% |
